# Asthma Is Associated with Multiple Alterations in Anti-Viral Innate Signalling Pathways

**DOI:** 10.1371/journal.pone.0106501

**Published:** 2014-09-09

**Authors:** Antonia L. Pritchard, Olivia J. White, Julie G. Burel, Melanie L. Carroll, Simon Phipps, John W. Upham

**Affiliations:** 1 Lung and Allergy Research Group, School of Medicine, The University of Queensland, Translational Research Institute (TRI), Woolloongabba, Brisbane, Australia; 2 School of Biomedical Sciences, The University of Queensland, Brisbane, Australia; 3 Department of Respiratory Medicine, Princess Alexandra Hospital, Brisbane, Australia; University of Hong Kong, Hong Kong

## Abstract

**Background:**

Human rhinovirus (HRV) infection is a major trigger for asthma exacerbations. Anti-viral immunity appears to be abnormal in asthma, with immune dysfunction reported in both airway structural cells and migratory, bone marrow derived cells. Though decreased capacity to produce anti-viral interferons (IFNs) has been reported in asthma, a detailed analysis of the molecular events involved has not been undertaken.

**Objective:**

To compare the molecular pathway controlling type I IFN synthesis in HRV-stimulated peripheral blood mononuclear cells (PBMC) from asthmatic and healthy subjects.

**Methods:**

PBMC from 22 allergic asthmatics and 20 healthy donors were cultured with HRV for 24 hours. Multiple components of the Toll-like receptor (TLR), IFN regulatory and NFκβ pathways were compared at the mRNA and protein level.

**Results:**

Multiple deficiencies in the innate immune response to HRV were identified in asthma, with significantly lower expression of IFNα, IFNβ and interferon stimulated genes than in healthy subjects. This was accompanied by reduced expression of intra-cellular signalling molecules including interferon regulatory factors (IRF1, IRF7), NF-κB family members (p50, p52, p65 and IκKα) and STAT1, and by reduced responsiveness to TLR7/TLR8 activation. These observations could not be attributed to alterations in the numbers of dendritic cell (DC) subsets in asthma or baseline expression of the viral RNA sensing receptors TLR7/TLR8. In healthy subjects, blocking the activity of type-I IFN or depleting plasmacytoid DC recapitulated many of the abnormalities observed in asthma.

**Conclusions:**

Multiple abnormalities in innate anti-viral signalling pathways were identified in asthma, with deficiencies in both IFN-dependent and IFN-independent molecules identified.

## Introduction

Respiratory viruses are associated with the majority of asthma exacerbations, which are a major cause of disease morbidity. Asthmatics do not appear to have more frequent viral infections than healthy individuals, but instead suffer more persistent and severe lower respiratory tract symptoms [1], [2]; human rhinovirus (HRV) infections are the most commonly identified in older children and adults. Given the importance of HRV in asthma and the paucity of effective anti-viral therapeutic options, a better understanding of the immune and inflammatory response to HRV is a significant focus of current respiratory research.

In response to HRV infection *in vitro*, a number of reports indicate that airway epithelial cells from people with asthma have a reduced capacity for innate interferon (IFN) synthesis, relative to normal airway epithelial cells [3], [4]. Deficient gene expression and/or synthesis of IFNα, IFNβ and IFNλ in epithelial cells and alveolar macrophages have been described in asthma [3], [4], although these findings have not been confirmed by some investigators [5], [6].

During acute infection it is a well-established paradigm that affected tissue sites signal the bone marrow and central lymphoid organs to recruit the immune cell populations required for pathogen neutralization. This process goes beyond mere chemo-attraction, and can include functional programming of migratory myeloid and lymphoid precursors within the bone marrow, prior to their arrival at mucosal surfaces [7]. These migratory immune cells represent an important reservoir during acute infection that supplements host defence provided by resident lung leukocytes. It is noteworthy in this regard that dysregulated anti-viral immune responses have been demonstrated in circulating populations of innate/adaptive immune cells in asthma [8]. PBMC from asthmatic children and adults secrete less IFNα following *in vitro* exposure to viruses [9], [10], which is associated with reduced function of Toll-like receptor (TLR)-7, a key receptor for single stranded viral RNA [11]; TLR3 function appears to be equivalent in asthmatic and healthy individuals. Notably, other investigators report that HRV-activated PBMC from people with mild or well controlled asthma exhibit normal function *in vitro* [12].

As plasmacytoid dendritic cells (pDC) are a potent source of type-I IFN synthesis during virus infections [13], some researchers have examined the role of pDC in asthma (recently reviewed [14]). Numerical changes in circulating pDC have been linked both to asthma development in young children [15] and to established asthma in adults [16]. The function of pDC also appears to be abnormal in asthma, with reports demonstrating that pDC from allergic asthmatics are less able to synthesise IFNα in response to influenza A [17] or TLR9 activation [18] than pDC from healthy subjects.

Little progress has been made in defining the detailed mechanisms by which HRV induces an innate immune response in migratory leukocytes, and which of these mechanisms are altered in asthma. Accordingly, the current study examined various processes associated with HRV stimulated IFN production, including recognition of viral RNA by TLRs, signalling pathways associated with the induction of IFNα and IFNβ gene expression, and molecules linked to viral entry. These data provide evidence that allergic asthma is associated with multiple alterations in anti-viral innate interferon signalling pathways. This appears to involve abnormalities in the regulation of TLR7, TLR8, interferon regulatory factors and pDC.

## Methods

### Study Cohorts

Healthy adult volunteers and allergic asthmatic volunteers were recruited. All subjects answered a questionnaire detailing symptoms of respiratory disease and had skin prick testing (SPT) to a panel of 10 common inhaled allergens (*Aspergillus fumigates*, Alternaria, Bahia, Couch grass, Ragweed, Southern grass, Ryegrass, Johnson, house dust mite and cat dander). All asthma volunteers had mild-to-moderate disease and had experienced asthma symptoms within the preceding 12 months; just over half were prescribed inhaled steroids and withheld this medication for 24 hours prior to blood sampling.

The study was approved by the Princess Alexandra Hospital and the University of Queensland Human Research Ethics Committees, and written informed consent was obtained from each subject.

### Rhinovirus generation and titration

HRV16 stocks were generated by passage in Ohio HeLa cells, as described previously [19] followed by purification over a sucrose gradient [20]. To define the optimal concentration of HRV, the TCID_50_ was determined as previously described [21].

### Cell culture

PBMC were isolated from heparinised blood by density gradient centrifugation and cultured at 1×10^6^ PBMC/mL of media, as previously described [11]. Stimuli included: HRV16 at a multiplicity of infection (MOI) of five, the TLR7/8 agonist gardiquimod (GQ; Invivogen, San Diego, CA), used at 0.3 µg/mL (TLR7 specific) and 5 µg/mL (TLR7/8 specific), and the TLR3-agonist polyinosine-polycytidylic acid (poly I:C; Invivogen, San Diego, CA), used at 25 µg/mL, (also activates protein kinase C and MAP3K7 [22]). Supernatant was harvested for cytokine quantification by ELISA and cell pellets were collected for RNA extraction using the RNeasy plus mini kit (Qiagen, Australia). B18R (Ebioscience, San Diego, CA) acts as a decoy receptor with high specificity and affinity for all known subtypes of the type-I IFN family, thereby blocking type-I IFN signalling into target cells [23].

### Time course of changes in gene expression and innate proteins

Detailed time course experiments were initially performed to assess gene expression at 0.5, 1, 3, 6, 12 and 24 hours after HRV exposure (Figure S1 in File S1); 24 hours post-HRV stimulation was chosen, based on these findings. The optimal time point for detection of IFNα protein and IFN-gamma-inducible protein 10 (CXCL10, also known as IP-10) by ELISA was previously shown at 24 hours [24].

### Depletion of peripheral plasmacytoid dendritic cells (pDC)

PBMC were depleted of pDCs using CD304 immuno-magnetic beads (Miltenyi Biotec, Germany). Cells were depleted using an AutoMACS according to the manufacturer’s instructions (Miltenyi Biotec, Germany). Purity of pDC depletions were assessed using flow cytometry and were found to be greater than 95% [21]. Control samples underwent “sham depletion” in which PBMCs were resuspended in buffer containing only FcR blocking reagent and no microbeads, prior to being run through the AutoMACS columns. Sham depleted and pDC depleted cultures were then either exposed to HRV stimulation or were unstimulated.

### ELISA

CXCL10 ELISA was performed using commercially available paired antibodies and recombinant cytokines (Becton Dickenson, Franklin Lakes, NJ); the limit of detection was 15.6 pg/ml. IFN-α (PBL Interferon Source, Piscataway, NJ) was assayed via commercial ELISA kit according to the manufacturer’s instructions; the IFN-α “multi-subtype” kit detects all isoforms except IFNα_13_ and IFNα_21_. The limit of detection was 9.7 pg/ml. Active Motif TransAM Kits and the Cayman Chemical NF-κB (human p50/p65) Combo Transcription Factor Assay Kits were used as per the manufacturers’ protocols.

### Quantitative Real Time PCR

RNA was reverse transcribed using Transcriptor first strand cDNA synthesis kit (Roche Diagnostics, Australia), according to manufacturer’s instructions. Gene expression was investigated by quantitative real time PCR (qPCR), using the LightCycler 480 (Roche Diagnostics, Australia). Data was analysed using the methodology described by Pflaffl et al [25]. *UBE2D2* was initially identified as a stable reference gene in CD4+ cells [26] and subsequently assessed in-house to be stably expressed in total PBMC in the absence and presence of HRV16, using the method described by Silver et al [27]. Table S1 shows the primers used to amplify IFNβ, myxovirus (influenza virus) resistance 1, interferon-inducible protein p78 (MxA; also known as Mx1), 2′,5′-oligoadenylate synthetase (OAS1), interferon regulatory factor (IRF) -1, -5, -7 and TLR-7, -8. The NF-κB subunits (p65, p52, p50, RELB, c-REL), IκKα, IκKβ, IκKε and IFNAR were assessed using ABI Taqman assays (Life Technologies, Australia). Results are expressed as a ratio of stimulated to control (unstimulated) samples, with a fold change of 1 representing unstimulated expression levels.

### Flow Cytometry

PBMC were washed with PBS 1% FCS (FACS Buffer) and stained for 20 min at 4°C with the following surface antibody cocktail: CD14-PerCp (clone MφP9; Becton Dickenson, Franklin Lakes, NJ), CD19-APCeF780 (clone SJ25C1; eBioscience, San Diego, CA), HLA-DR-APC (clone G46-6; Becton Dickenson), CD123-FITC (clone 6H6; Biolegend, San Diego, CA) and CD1c-PE/Cy7 (clone L161; Biolegend). Cells were fixed and permeabilised for 10 min using the BD Fix/Perm solution 1× (Becton Dickenson) at 4°C, then washed with BD Perm/Wash 1× buffer (Becton Dickenson), and stained with TLR7-PE dilution 1/50 (polyclonal; Abcam, Cambridge, UK), TLR8-PE dilution 1/50 (clone 44C143; Abcam), IRF7-PE dilution 1/50 (clone K40-321; Becton Dickenson) or ICAM1-PE dilution 1/50 (clone HA58; Becton Dickenson) for 30 min at 4°C. Cells were then washed with FACS buffer, resuspended in PFA 4% and kept at 4°C until acquisition. Acquisition was performed on a BD FACS Canto cytometer (Becton Dickenson) with DIVA 2.0 software (Dialogic, Montreal, Canada). An average of 800,000 events, gated on the lymphocyte/monocyte population, was acquired for all experiments; the gating strategies are shown in Figure S2 in File S1. Data were analysed with FlowJo software version 7.6 (Ashland, OR).

### Statistics

Statistical analysis was performed using Graphpad Prism 5 for Windows (GraphPad Software, San Diego, CA). The data was not normally distributed, so results are presented as medians and interquartile ranges and analysed non-parametrically using the Mann-Whitney U test.

## Results

Subjects included in this study comprised twenty-two allergic asthmatics and twenty non-atopic healthy controls (Table 1). Given our previous findings that anti-viral immunity varies with sex [24], we ensured a balanced distribution of women and men in each study group. None of the study cohort were current smokers and any asthma patient taking inhaled steroids withheld use for 24 hours prior to blood draw. Allergic sensitisation was significantly more prominent in the asthma group than in the healthy group, but in other respects, including age and BMI, both groups were well matched (Table 1).

**Table 1:** Demographics of healthy control and asthma patient cohorts.

Characteristic	Healthy Cohort	Asthma Cohort	p
N	20	22	n/a
Sex	50% female	50% female	n/a
Mean Age (±SD)	35.3 years±12.6	33.83 years±12.9	0.91
Mean total SPT (±SD)*	0	20 mm±11	<0.001
Mean number of positive SPT (±SD)*	0	4.47±2.39	<0.001
Mean wheal diameter/positive SPT (±SD)	0	4.93 mm±1.89	<0.001
Mean Body Mass Index (±SD)	23.97±3.69	25.67±4.01	0.24
Taking inhaled steroids	0	12 (54%)	n/a

*Calculated from 10 common allergens; Aspergillus fumigates, Alternaria, Bahia, Couch grass, Ragweed, Southern grass, Ryegrass, Johnson, Dust mite (DPT) and cat dander.

**Figure 1:**
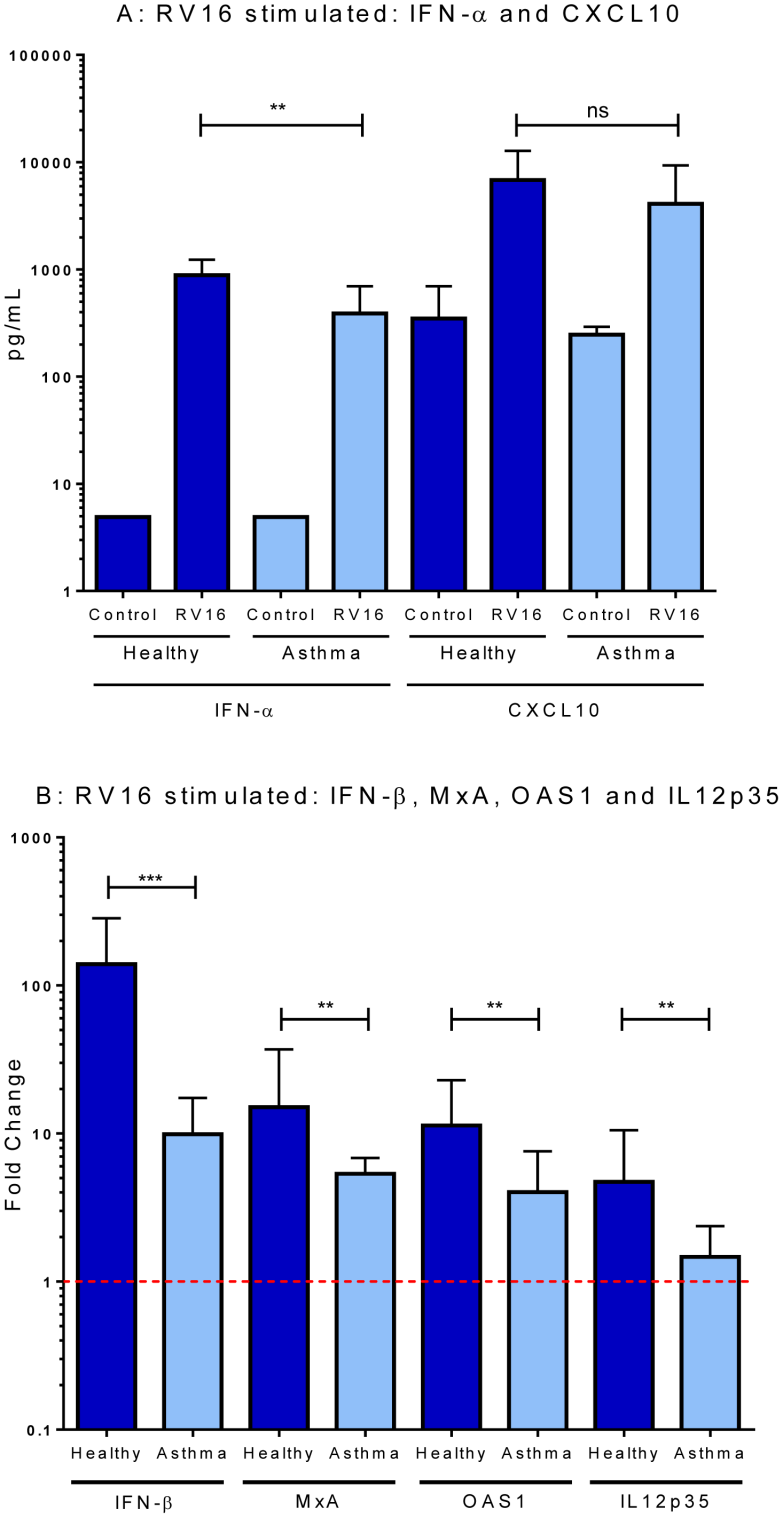
Innate responses to HRV16. PBMC derived from healthy controls and asthmatic patients were stimulated with HRV16 at an MOI = 5 for 24 hours. IFNα was measured in cell culture supernatants by ELISA (A) Expression of IFNβ, MxA, OAS1, and IL12p35 was measured by qPCR of cell extracts (B) and are expressed as the fold change in gene expression in stimulated cells, which is normalised to unstimulated cultures; the dotted line at 1 represents no change in gene expression from the unstimulated cultures [25]. Data are displayed as median and IQR. ns: not significant, **p value <0.01, ***p value <0.001 using Mann-Whitney *U*-test comparing healthy (n = 20) to asthmatic (n = 22).

We undertook a detailed examination of the innate immune response to HRV16 in allergic asthmatic and healthy control subjects. HRV exposed PBMC from asthmatics produced significantly less IFNα proteins than PBMC from healthy control subjects (median 388 pg/mL vs. 881 pg/mL, p<0.01; Figure 1A). In contrast, synthesis of the chemokine CXCL10 was similar in asthmatic and control subjects (Figure 1A). Relative to control subjects, PBMC from the asthmatic group also showed significantly lower expression of *IFNβ,* the IFN-responsive genes *MxA* and *OAS1*, and the Th1-polarising cytokine *IL-12p35* (Figure 1B). We have been previously unable to detect IFNλ production by PBMC in response to HRV, using either qPCR or ELISA [21], so this was not assessed. When PBMC were stimulated with a low concentration of GQ (0.3 µg/mL), known to elicit a low TLR7 specific response, only a slight IFNα response was observed in both healthy controls and asthmatics (11.5 pg/mL±12.2 and 4.9 pg/mL±27.7, respectively) and no difference was shown between healthy and asthmatic subjects in *IFNβ* mRNA expression (8.03±16.3 vs 8.34±24.3, p>0.05). Only with a higher concentration of GQ (5 µg/mL) that robustly activates both TLR7 and TLR8 did differences emerge between groups, with significantly lower *IFNβ* expression in cells from asthmatics than in cells from healthy controls (2.20±3.4 vs. 5.86±7.3, respectively p<0.05). Stimulation of PBMC by the TLR3 agonist polyI:C revealed no difference in IFNβ expression between cells derived from asthmatics compared to healthy controls (31.34±80.53 vs. 47.63±78.05, respectively p>0.05), supporting our previous findings [11].

**Figure 2:**
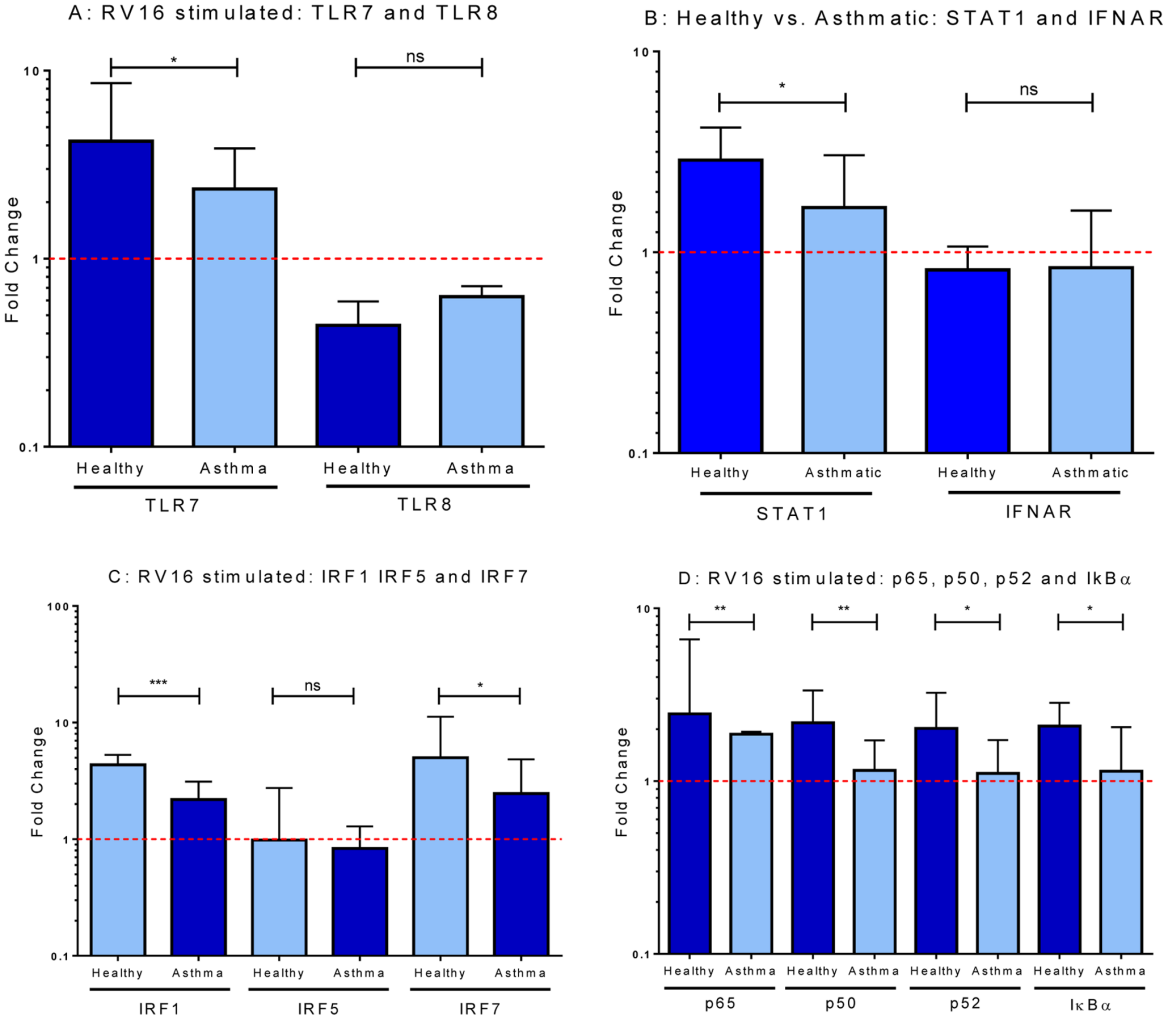
HRV16-induced expression of genes associated with the innate signalling pathways in PBMC from healthy controls and asthmatics. PBMC derived from healthy controls and asthmatic patients were stimulated with HRV16 (MOI = 5) for 24 hours. mRNA expression of TLR7 and TLR8 (A), STAT1 and IFNAR (B), interferon regulatory factors IRF1, IRF5, and IRF7 (C) and NFκB subunits p65, p50, p52, and IκBα (D) were measured by qPCR. Results are displayed as the fold change in gene expression in stimulated cells, which is normalised to unstimulated cells; the dotted line at 1 represents no change in gene expression from the unstimulated cultures [25]. Data are displayed as median and IQR. ns: not significant, *p value <0.05, **p value <0.01 using Mann-Whitney *U*-test comparing healthy (n = 20) to asthmatic (n = 22).

We next investigated TLRs that detect viral ssRNA together with key signalling molecules involved in anti-viral innate immunity. HRV induced up-regulation of TLR7 mRNA expression in both groups, though the magnitude of the increase was significantly less in asthmatic subjects (p<0.05, Figure 2). In contrast, HRV induced down-regulation of TLR8 mRNA expression, which occurred to a similar extent in both cohorts (Figure 2). Three interferon regulatory factors were also examined because of the role they play in type I IFN regulation. *IRF1* and *IRF7* expressions were lower in asthmatic subjects than in healthy subjects following HRV stimulation (p<0.01 and p<0.05, respectively, Figure 2), whereas *IRF5* mRNA expression was not altered by HRV stimulation in either group (p = non-significant; Figure 2). HRV-induced signal transducer and activator of transcription-1 (*STAT1*) expression was significantly lower in asthmatic subjects than in control subjects (p<0.05; Figure 2), though HRV did not alter mRNA expression of *IFNAR* (the common receptor for IFN-α and IFN-β) in either control or asthmatic subjects (Figure 2). HRV also induced changes in multiple NF-κB associated molecules as detailed in Figure S1A in File S1. The mRNA expression of *p65*, *p50*, *p52* and *IκKα* were chosen for more detailed assessment: all showed significantly lower expression in asthmatic subjects than in control subjects (*p65* and *p50* p<0.01, *p52* and *IκKα* p<0.05; Figure 2). While there are ELISA-based methods available to assess nuclear-translocated (active) NF-κB transcription factors p65 and p50 in cell lines, we found that neither colourimetric nor chemiluminescence assays could reliably detect these proteins in our experimental model i.e. primary cultures of human PBMC stimulated with HRV (*data not shown*). Extensive but unsuccessful attempts were also made to measure the activated (phosphorylated) NF-κB subunit p65 and IRF7 using flow cytometry, but it was not possible to reliably detect phosphorylated p65 and IRF7 over and above background staining.

**Figure 3:**
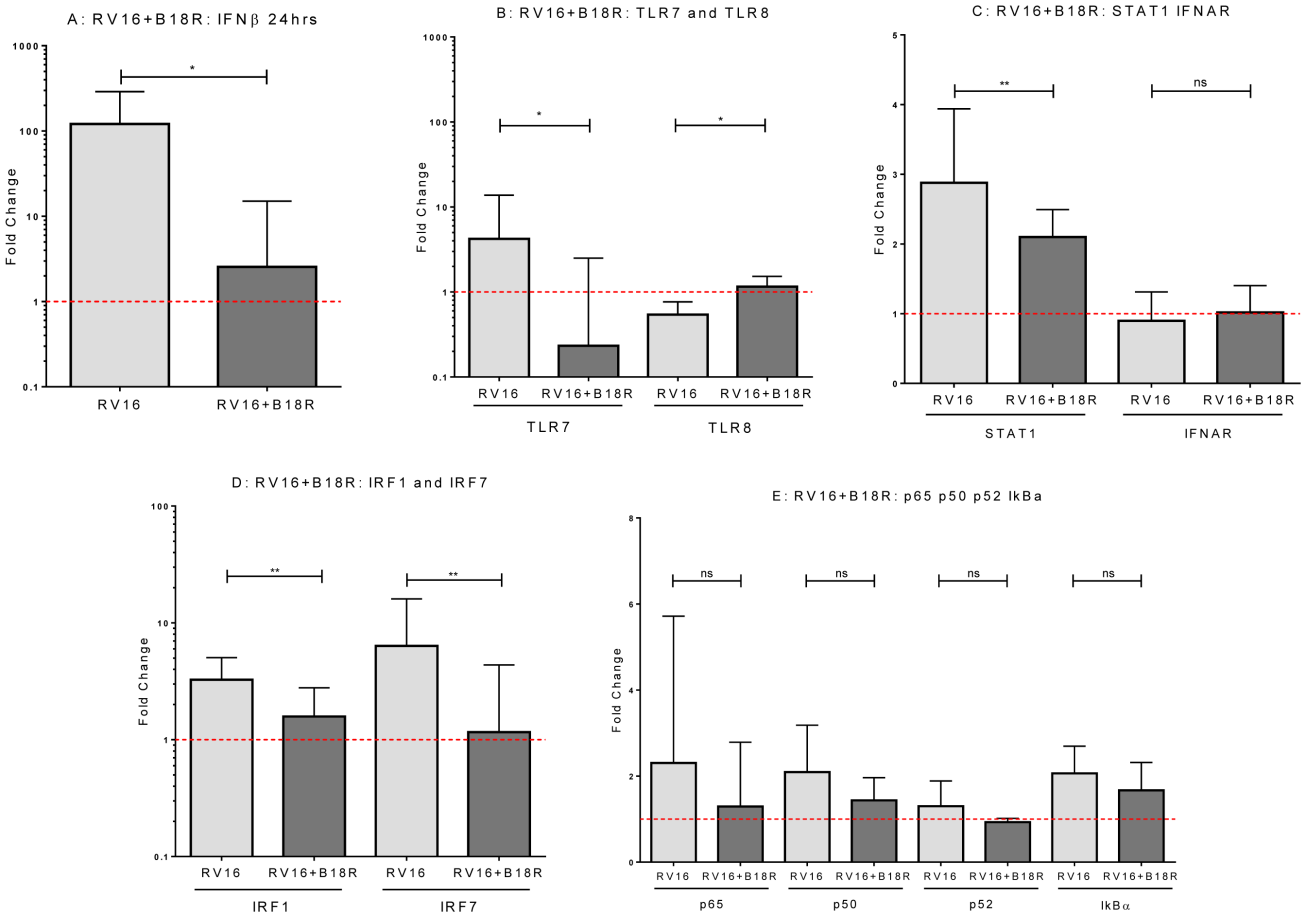
HRV16-induced expression of genes associated with the innate signalling pathways in PBMC pre-treated with the IFNAR blocking agent/decoy receptor B18R. PBMC derived from healthy controls were pre-treated with B18R (0.1 µg/mL) for 1 hour prior to stimulation with HRV16 (MOI = 5) for 24 hours. mRNA expression of IFNβ (A) TLR7 and TLR8 (B), STAT1 and IFNAR (C), interferon regulatory factors IRF1 and IRF7 (D) and, NFκB subunits p65, p50, p52, and IκBα and (E) were measured by qPCR. Results are displayed as the fold change in gene expression in stimulated cells, which is normalised to unstimulated cells; the dotted line at 1 represents no change in gene expression from the unstimulated cultures [25]. Data are displayed as median and IQR. ns: not significant, *p value <0.05, **p value <0.01 using Mann-Whitney *U*-test comparing healthy (n = 20) to asthmatic (n = 22).

We next sought to determine whether manipulating type I IFNs and pDC in cultures from healthy subjects might recapitulate the impaired responses to HRV observed in asthma. When B18R (a competitive inhibitor of the bioactivity of innate IFNs), was added to HRV-stimulated cells from healthy subjects, it significantly inhibited the induction of *IFNβ* transcription (p<0.05; Figure 3), consistent with the known capacity of type-I IFNs to stimulate their own expression and production. B18R also suppressed HRV induced *TLR7* mRNA (p<0.05; Figure 3), *IRF1* and *IRF7* (p<0.01, p<0.01, respectively) and inhibited HRV induced down-regulation of *TLR8* mRNA expression (p<0.05; Figure 3). B18R inhibited STAT1 upregulation (Figure 3), but had no effect on IFNAR expression (Figure 3D), and no effect on mRNA expression of *p65*, *p50*, *p52* and *IκKα* (Figure 3).

**Figure 4:**
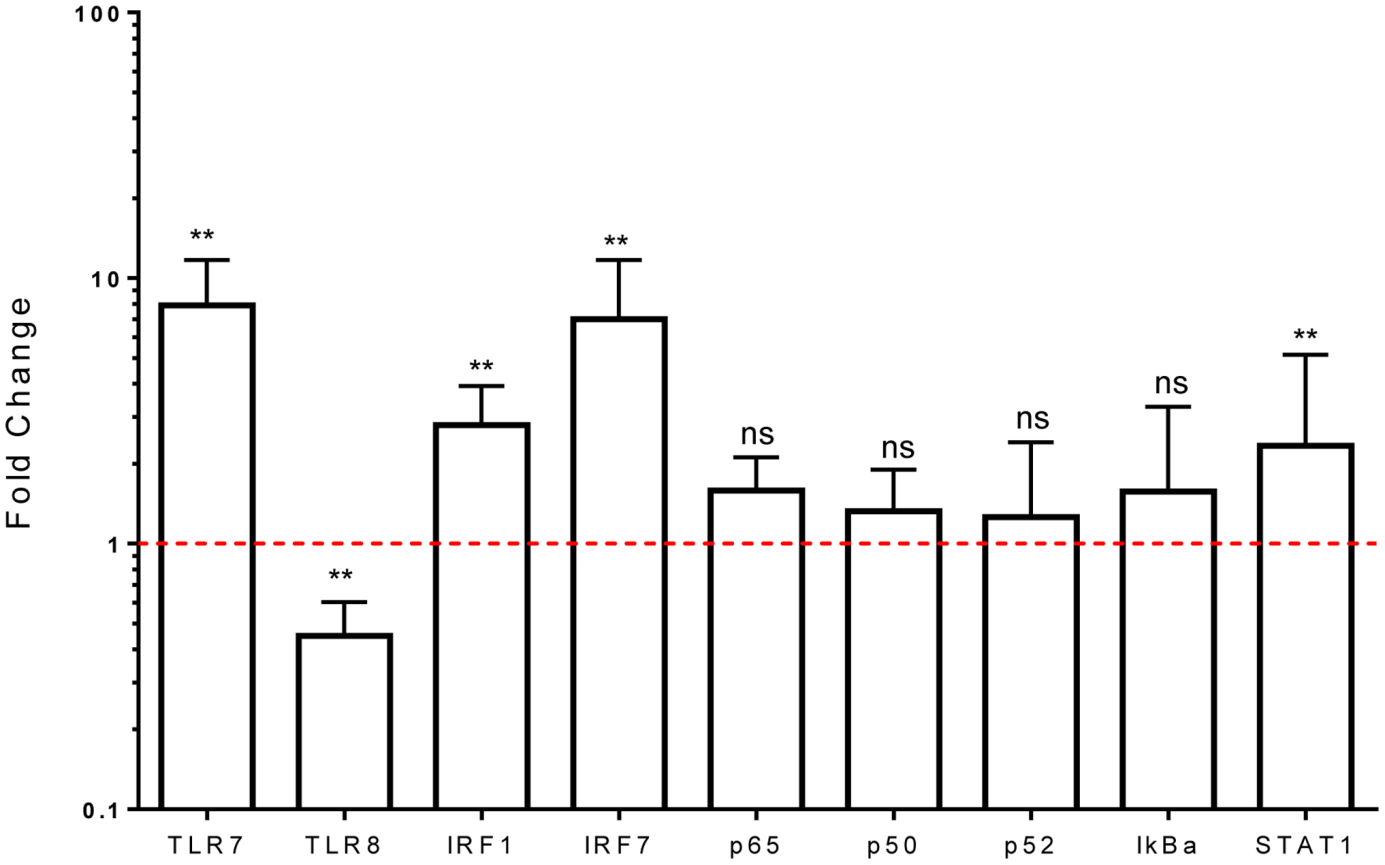
Innate signalling molecule responses to IFNβ alone. PBMC derived from healthy controls were exposed to IFNβ 30 ng/ml for 24 h in the absence of virus. mRNA expressions of TLR7, TLR8, IRF1, IRF7, and the NFκB subunits p65, p50, p52, and IκBα were measured by qPCR. Results are displayed as the fold change in gene expression in IFNβ stimulated cells, which is normalised to unstimulated cells; the dotted line at 1 represents no change in gene expression from the unstimulated cultures [25]. Data are displayed as median and IQR and the difference between IFNβ stimulated and unstimulated was statistically examined. ns: not significant, *p value <0.05, **p value <0.01.

Addition of recombinant IFNβ induced similar CXCL10 secretion in control and asthmatic subjects (Figure S4 in File S1), confirming earlier reports that cells from asthmatics have normal responses to IFNβ stimulation [29]. Exposing healthy PBMC to recombinant IFNβ in the absence of HRV16 led to significant induction of *TLR7*, *IRF1*, *IRF7* and *STAT1* expression and down-regulation of *TLR8* (Figure 4), indicating that these genes are indeed IFN responsive. In contrast, the NF-κB subunits *p65*, *p50*, *p52* and *IκKα* did not appear to be responsive to IFNβ (Figure 4).

**Figure 5:**
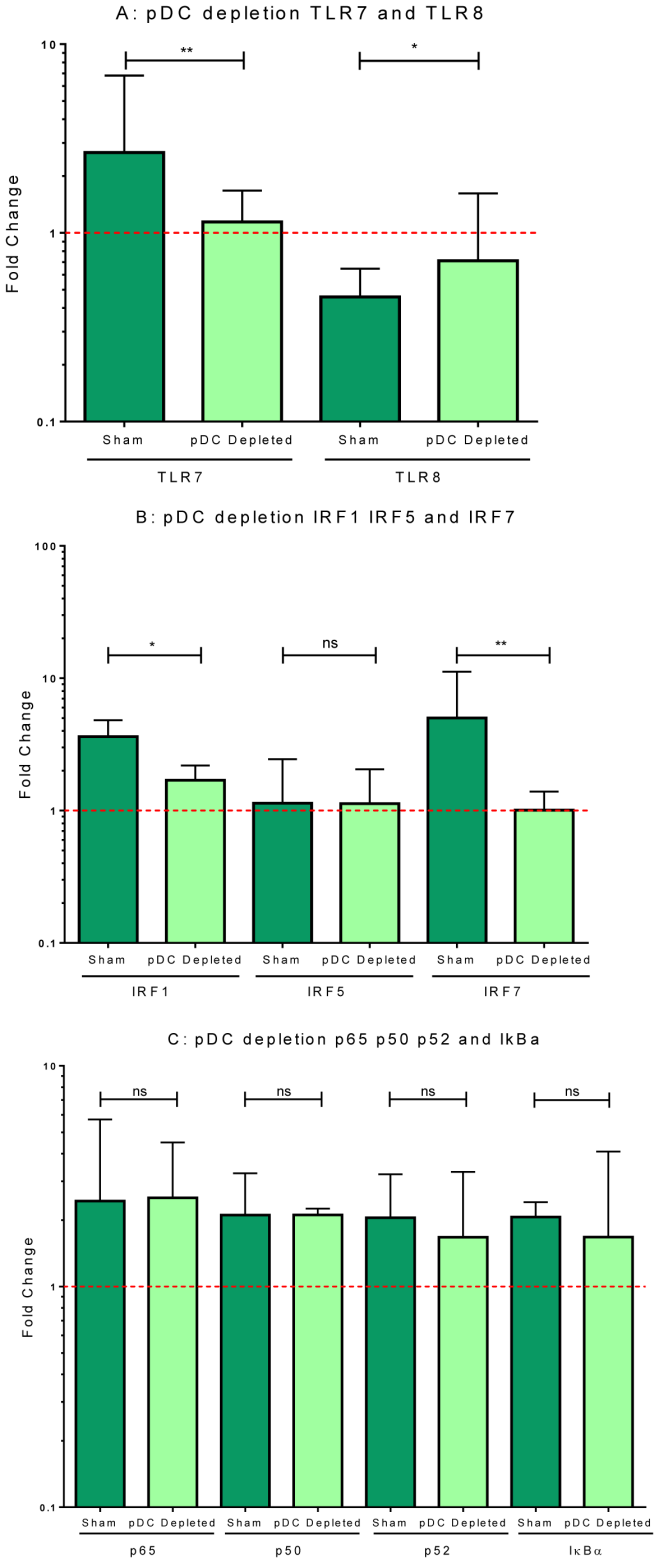
HRV16-induced expression of genes associated with the innate signalling pathways in PBMC depleted of pDC. PBMC derived from healthy controls were depleted of pDC by AutoMacs using CD304 monoclonal antibody or no antibody (Sham) and then stimulated with HRV16 (MOI = 5) for 24 hours. mRNA expression of TLR7 and TLR8 (A), interferon regulatory factors IRF1, IRF5, and IRF7 (B), and NFκB subunits p65, p50, p52, and IκBα (C) was measured by qPCR. Results are displayed as the fold change in gene expression in stimulated cells normalised to unstimulated cells; the dotted line at 1 represents no change in gene expression [25]. Data are displayed as median and IQR. ns: not significant, *p value <0.05, **p value <0.01 using Mann-Whitney *U*-test comparing sham depleted (n = 10) to pDC depleted (n = 10) cultures.

We then investigated the role of pDC in this model, by depleting them from the cultures; we have previously shown that pDC are responsible for >98% of IFNα secretion in HRV16 stimulated PBMC [21]. In healthy control subjects, depletion of pDC led to a similar pattern of gene expression as that seen with B18R: significant alterations in *TLR7*, *TLR8*, *IRF1*, *IRF7* expression, but no change in NF-κB subunit expression (Figure 5A, 5B and 5C). Limited amounts of available RNA precluded assessment of *STAT1* and *IFNAR* expression in these experiments.

**Figure 6:**
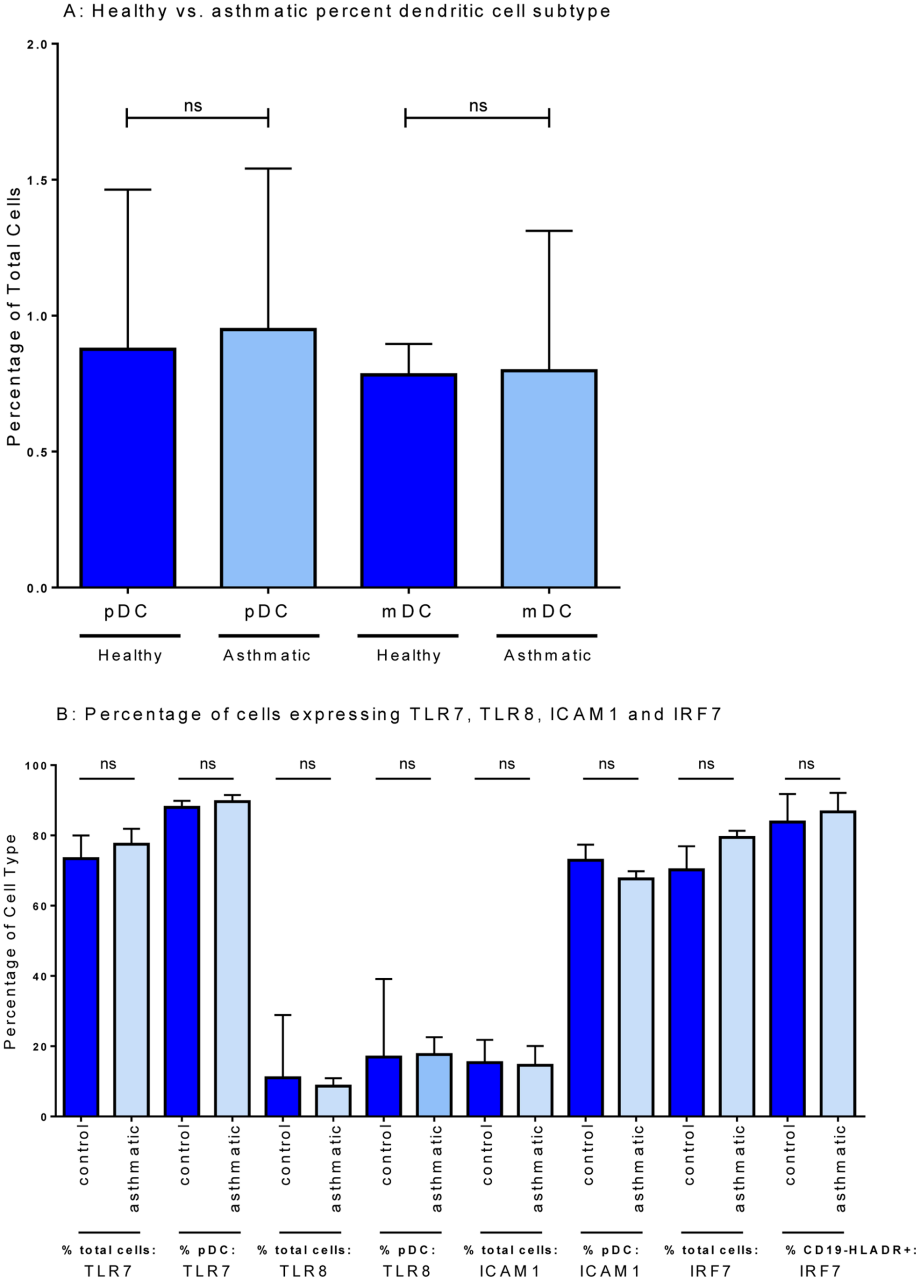
Proportion of dendritic cell subsets in PBMC from healthy controls and asthmatics and expression of TLR7, TLR8, ICAM1, and IRF7. Unstimulated PBMC were stained with fluorescent-labelled antibodies as stated in methods. The percentage of plasmacytoid DC (pDC: CD14− CD19− HLADR+ CD1c− CD123+), and myeloid DC (mDC: CD14− CD19− HLADR+ CD1c+ CD123−) are displayed as median and IQR comparing healthy and asthmatic (A). The percentage of total PBMC and pDC expressing TLR7, TLR8, ICAM1, and IRF7 by intracellular staining are displayed (B). ns: not significant using Mann-Whitney *U*-test comparing healthy (n = 20) to asthmatic (n = 20).

It was possible that the deficiencies in type I IFN and IFN-associated genes observed in asthma (Figures 1 and 2) might be attributed to baseline differences in key cell populations, or expression of receptors responsible for detecting viral ssRNA prior to stimulation. The relative proportions of circulating pDC and mDC were similar in asthmatic and control subjects (Figure 6A), as were the proportions of CD19+ B-cells and CD14+ monocytes (*data not shown*). Expressing HRV-stimulated IFNα secretion relative to the proportion of circulating pDC in the cultures, indicated that pDC from healthy subjects secrete approximately two-fold more IFNα on a per cell basis than asthmatics. The proportion of cells staining for ICAM-1 (the entry receptor for major group HRVs), TLR7 and TLR8 prior to stimulation was identical in asthmatic and control subjects, in total PBMC and in pDC (Figure 6B). TLR7 was expressed in the majority of monocytes, pDC and mDC, while TLR8 was more frequently present in monocytes than in pDC and mDC (Figure S3A and 3B in File S1). Back-gating on the TLR7 or TLR8 positive cells (gating strategy shown in Figure S2 in File S1) revealed that the proportions of cell types measured by our FACS panel within PBMC did not differ between the control cohort and the asthmatic cohort (Figure 6A; Figure 6B). We also examined intracellular non-phosphorylated IRF7, a signal transduction protein that is important for TLR signalling and the regulation of type-I IFN expression [28]. Although technical limitations with the staining protocol prevented assessment of IRF7 specifically in pDC, baseline (unstimulated) expression of IRF7 in unstimulated HLADR+CD19− cells (which includes pDC, mDC and monocytes) was similar in asthmatic and healthy control subjects (Figure 6B).

## Discussion and Conclusions

In this study we performed a detailed analysis of HRV-stimulated innate immune responses *ex-vivo*, using circulating immune cells from allergic asthmatic and healthy donors. Our aims were to determine the extent to which HRV-induced gene expression is dependent on type I IFN and pDC, and to compare response patterns in asthmatic and healthy donors.

By employing a variety of experimental approaches (blocking type I IFN bioactivity, addition of recombinant IFNβ, and pDC depletion), we were able to confirm that the ability of HRV to enhance *TLR7*, *IRF1*, *IRF7* and *STAT1* expression is dependent on type-I IFN and pDC in cultured cells from healthy donors. HRV also induced *TLR8* down-regulation in a type-I IFN dependent manner. This is an interesting observation that does not appear to have been previously reported. The functional consequences of *TLR8* inhibition during HRV infection are currently unknown, but this may alter IL-12 production, which was also observed to be differentially expressed between healthy controls and asthmatics, in response to HRV16 (see Figure 1) and merits further investigation. In contrast, the NF-κB family members *p50*, *p52*, *p65* and *IκKα* appear independent of type-I IFN and pDC. *IFNAR* expression also seems independent of type-I IFN, but insufficient RNA precluded assessment of whether *IFNAR* expression is regulated by pDC.

Multiple differences in innate immune responses were identified in asthmatic relative to healthy donors after HRV stimulation, including significantly lower expression/synthesis of type-I IFN and reduced expression of *TLR7,* the interferon stimulated genes *MxA* and *OAS1,* and *IL-12p35*. This was accompanied by reduced expression of intra-cellular signalling molecules including interferon regulatory factors (*IRF1*, *IRF7*), *STAT1* and several members of the NF-κB family (*p50*, *p52*, *p65* and *IκKα*). In contrast, expressions of *TLR8*, *IRF5* and *IFNAR* were similar after HRV stimulation in cells from asthmatic and healthy donors. These observations could not be attributed to alterations in the numbers of antigen presenting cells, or expression of ICAM-1, TLR7 or TLR8 at baseline, prior to HRV exposure.

Many investigators have proposed that a dysregulated innate immune response to respiratory viruses such as HRV is an important feature of asthma, though there is relatively little understanding of the mechanisms involved. Our findings confirm and extend previous reports that circulating immune cells (both PBMC and isolated pDC) from people with asthma have a lower capacity to express type-I IFNs or IFN-related genes [9], [10]. This is in contrast to the recent report of Sykes et al, who recently reported reductions in HRV-induced IFNα and IFNβ in well-controlled asthma were largely confined to lung cells, with no differences observed between PBMC from asthmatic and healthy donors [12]. The differences observed between our findings and those reported by Sykes et al could be due to phenotypic differences between study cohorts, including inflammatory phenotype, asthma severity and asthma control [12]. Variations in the degree of atopy, FcεR1 expression and extent of recent allergen exposure are also plausible reasons for variations in findings between different laboratories. FcεR1 cross-linking on peripheral blood pDC impairs the capacity to mount an anti-viral response [17]. Deficiencies in the capacity of HRV-stimulated PBMC to secrete type-I IFN in asthmatic children were most evident after cross-linking FcεR1 [30] and deficits in the ability of patients with allergic rhinitis to secrete IFNα have been described in pDC from both the nasal mucosa and peripheral blood [31]. More prosaic experimental factors such as virus strain and concentration, and the capacity of different assays to measure multiple IFNα subtypes may also be relevant.

Previous reports of deficient type I IFN synthesis from circulating cells in asthma have nearly always used RNA viruses such as Newcastle virus and RSV [9], [10], the influenza virus [17] and HRV [30]. This suggested to us that receptors for viral RNA, and/or their associated adaptor proteins warranted further study. HRV and other RNA viruses replicate in epithelial cells and other structural cells, so cytosolic receptors such as MDA5/RIG-I assume a major role in RNA detection in these cell types [32]. In contrast, viruses do not replicate in pDC and some other migratory leukocyte populations [33], [34] and viral RNA is instead detected by endosomal receptors such as TLR3, TLR7 or TLR8. We previously reported that asthma is associated with abnormal responsiveness to imiquimod (a mixed TLR7/TLR8 ligand), whereas TLR3 function was normal [11]. In the current study we employed GQ: at low concentrations this is specific for TLR7 but at higher concentrations both TLR7 and TLR8 are stimulated. Interestingly, differences between asthmatic and healthy subjects only became apparent at the higher concentration of GQ. TLR3 function was again normal, confirming our previous report [11]. Future studies are now clearly warranted to dissect the relative importance of TLR7 and TLR8 in asthma, and how these receptors interact, particularly given evidence from genetic association studies implicating both *TLR7* and *TLR8* gene variants in susceptibility to asthma [35] and allergic rhinitis [36].

It is noteworthy that blocking the activity of type I IFNs and depletion of pDC in cultured cells from healthy subjects recapitulated many of the abnormalities observed in the asthmatic donors. This provides strong circumstantial evidence that the altered innate immune response to HRV in allergic asthma can be partly attributed to reduced type-I IFN production and/or pDC dysfunction. There is a need for more detailed studies of the function of purified pDC from people with asthma, though the small numbers of available cells restricts the number of outcomes that can be evaluated in any one experiment.

Interestingly, it seems that asthma is also associated with altered IFN-independent immune pathways as exemplified by reduced expression of several NF-κB family members after HRV exposure (Figure 2).

Following viral entry into cells, type-I IFN synthesis and the induction of an anti-viral state within the cell follows a biphasic time course as shown in Figure S1 in File S1. Early synthesis of IFNα and IFNβ is followed by engagement of their common receptor (IFNAR), leading to a positive feedback loop that amplifies further synthesis of IFNα and IFNβ. Because the majority of our outcomes were measured at 24 h, it is uncertain whether the altered responses to HRV seen in asthma can be attributed to early events after viral detection, or are related to a failure of the IFN-driven positive feedback loop. Responsiveness to IFNβ and IFNAR expression appear similar in asthmatic and healthy donors, so we propose that very early events in the response to HRV may be critical in asthma; this may involve the subtle increases in gene expression noted at the early time points (Figure S1 in File S1), or the function of existing proteins. It is clear that examining these in some detail should be a focus of future research.

There are a number of potential limitations of this study that warrant comment. Firstly, while patients withheld medication for 24 hours prior to blood collection and the doses employed were unlikely to lead to systemic absorption, approximately half the asthma patients were being treated with inhaled corticosteroids. However, we observed similar deficiencies in innate immune function between those asthmatics taking inhaled corticosteroids and those who were not (Figure S5 in File S1), so we do not think that medication use adequately explains the findings outlined in Figures 1 and 2. Secondly, we studied HRV16, a relatively ‘benign’ laboratory-adapted strain of the virus and different findings may be obtained with more virulent HRV strains. Thirdly, the methodologies currently available to investigate innate immune response signalling molecules have several limitations, meaning that key endpoints, such as protein phosphorylation, could not be reliably assessed. Finally, our current experiments examined atopic asthmatics, and our findings, in combination with other recent studies [17], [32], suggest that comparison with non-atopic asthmatics could yield interesting findings.

Our findings shed light on the pathogenesis of virus-induced asthma exacerbations. In the setting of a viral upper respiratory tract infection, the deficiencies in innate immune pathway are likely to lead to an increased viral load, exaggerated lower airway inflammation and exacerbation of asthmatic symptoms. We have recently shown that another important consequence of decreased innate IFN production is an increase in T_H_2 cytokine synthesis by virus-specific memory T-cells [21], [37] that might intensify pre-existing T_H_2 mediated airway inflammation during HRV infection. Whether or not low IFN production and/or pDC dysfunction also contribute to a failure of immune regulatory mechanisms is currently under investigation. Taken together, our findings emphasise that decreased type-I IFN production has important consequences to patients and elucidation of the mechanisms behind this should be a key focus of research in the asthma field.

## Supporting Information

Table S1 Primer sequences for examination of gene expression by qPCR*.
(DOCX)

File S1 Contains figs. S1–S5.
(DOCX)
