# Supplementary material for: Asthma Is Associated with Multiple Alterations in Anti-Viral Innate Signalling Pathways
Source: PLoS One. 2014 Sep 9;9(9):e106501. doi: 10.1371/journal.pone.0106501 (PMC4159236; doi:10.1371/journal.pone.0106501)
Supplement: Table S1 — Primer sequences for examination of gene expression by qPCR*. (DOCX) [file pone.0106501.s001.docx]

**Supplementary Table 1: Primer sequences for examination of gene expression by qPCR*.**

| **Gene** | **Forward Primer Sequence** | **Reverse Primer Sequence** |
| --- | --- | --- |
| UBE2D2 | ATGGCAGCATTTGTCTTGATATTCTAC | TGGATTGGGATCACACAACAGA |
| IFNβ | CATTACCTGAAGGCCAAGGA | CAGCATCTGCTGGTTGAAGA |
| MxA | CTCGGCAACAGACTCTTCCAT | CATGAAGAACTGGATGATCAAAGG |
| OAS1 | AGAAATACCCCAGCCAAATCTCT | TGAGGAGCCACCCTTTACCA |
| TLR7 | ATGGTGTTTCCAATGTGGAC | GTTCGTGGGAATACCTCCAG |
| TLR8 | TCCTTCAGTCGTCAATGCTG | CGTTTGGGGAACTTCCTGTA |
| IRF1 | ACCCTGGCTAGAGATGCAGA | GTGTGAATGGCCCAGCTC |
| IRF5 | TTATTCTGCATCCCCTGGAG | GCTCTTGTTAAGGGCACAGC |
| IRF7 | GGCTGGAAAACCAACTTCC | GCCTCTGCCTCAGTCTGGT |
| TAK1 | TAACAAGGGGAGTGCTGCTT | GAACAGCCCACATGATTCG |
| Stat-1 | GGCAGTTTTCTTCTGTCACCA | GTCCACGGAATGAGACCATC |

* Note: The NF-κB subunits (p50, p52, p65, c-rel, REL-B), IκBα and IFNAR were assessed using ABI Taqman predesigned assays (Life Technologies, Australia).
