# Supplementary material for: Asthma Is Associated with Multiple Alterations in Anti-Viral Innate Signalling Pathways
Source: PLoS One. 2014 Sep 9;9(9):e106501. doi: 10.1371/journal.pone.0106501 (PMC4159236; doi:10.1371/journal.pone.0106501)

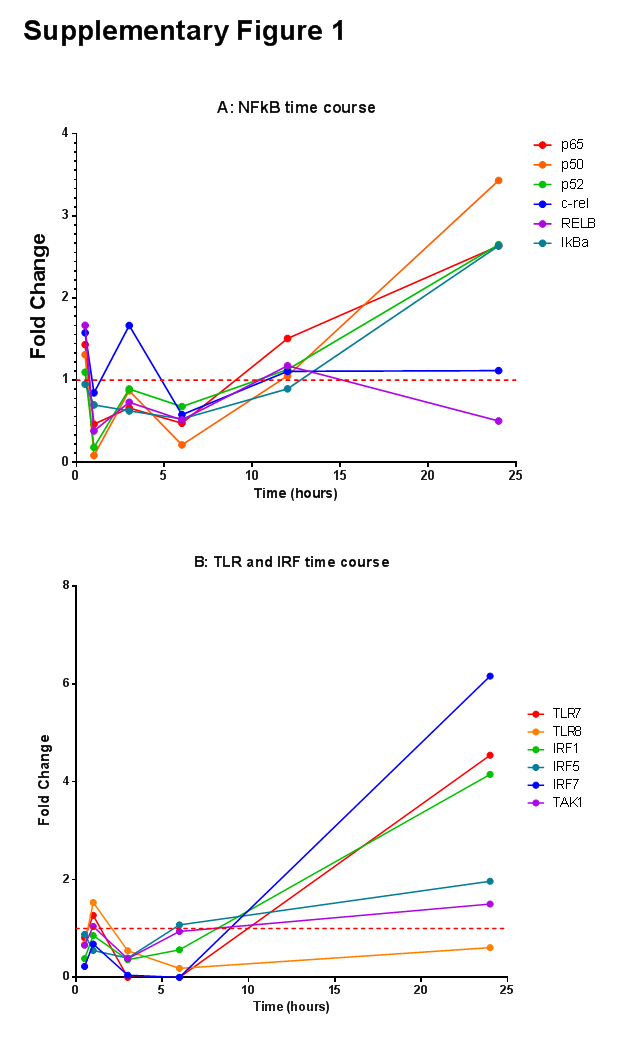


**Supplementary Figure 2: Gating strategies used in FACS analysis of PBMC**

A:

B:

**TLR7 backgating**:

1^st^ gate on TLR7+ cells    > CD14+ cells: **TLR7+ Monocytes**

> CD19+CD14- cells: **TLR7+ B cells**

> CD19-CD14- cells > HLADR+ cells > CD123+CD1c- cells: **TLR7+ pDCs**

> CD123-CD1c+ cells: **TLR7+ mDCs**

**TLR8 backgating**:

1^st^ gate on TLR8+ cells    > CD14+ cells: **TLR8+ Monocytes**

> CD19+CD14- cells: **TLR8+ B cells**

> CD19-CD14- cells > HLADR+ cells > CD123+CD1c- cells: **TLR8+ pDCs**

> CD123-CD1c+ cells: **TLR8+ mDCs**


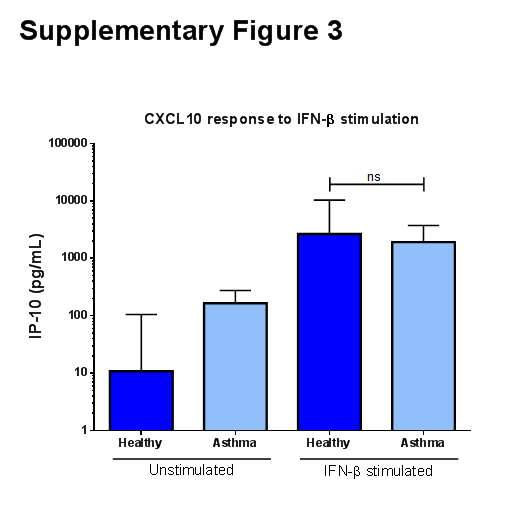


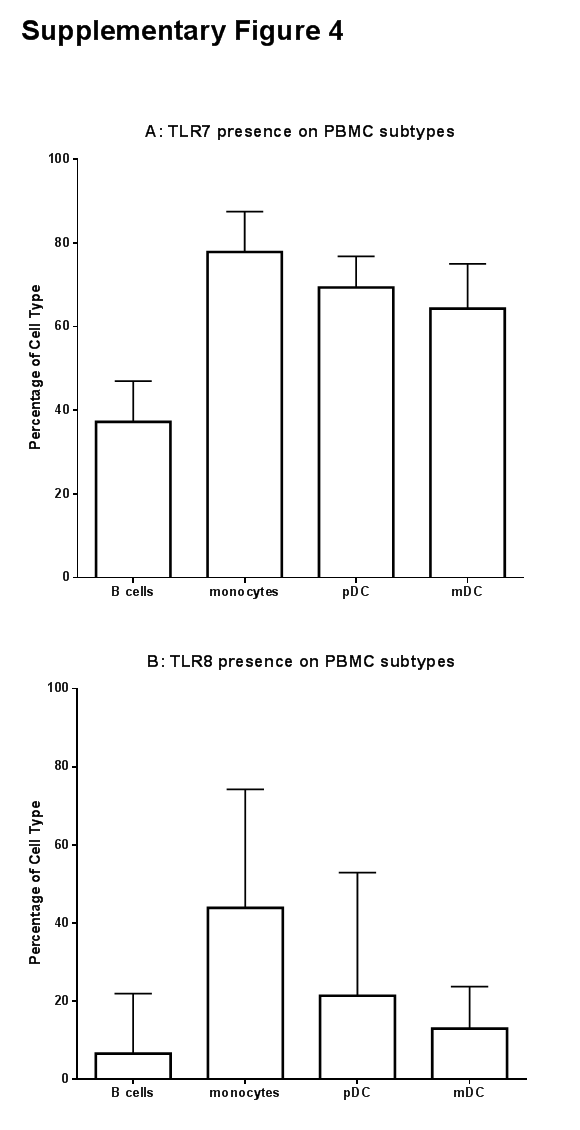


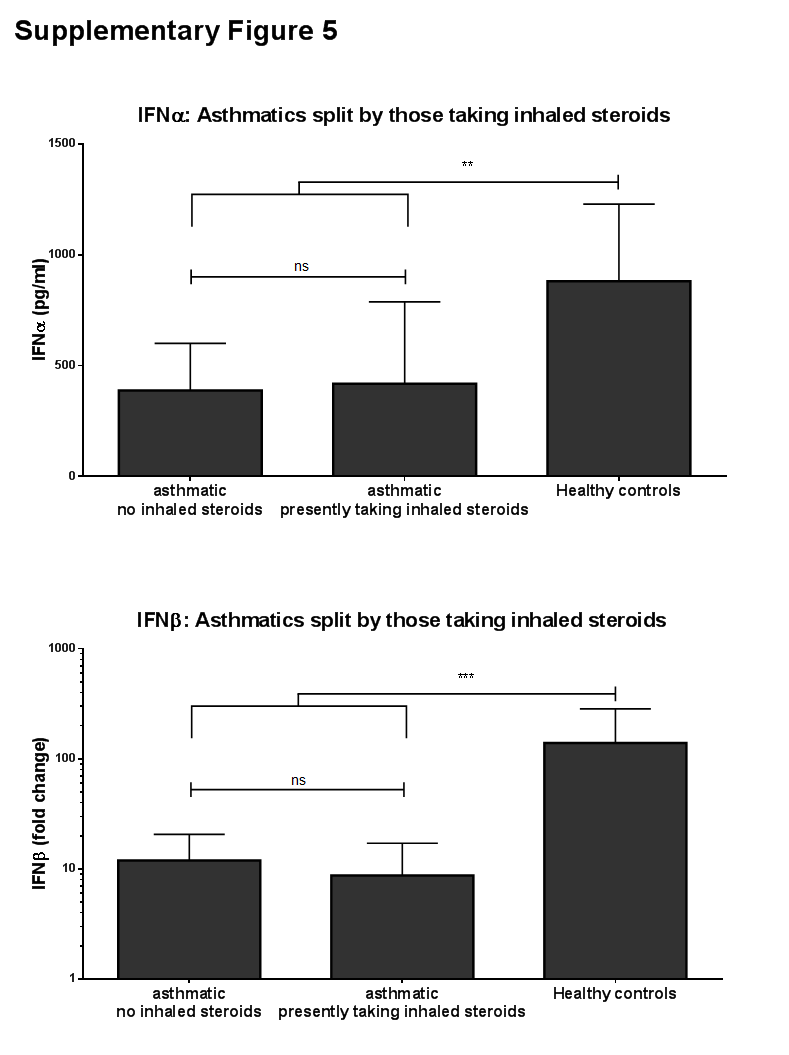

Supplement: File S1 — Contains figs. S1–S5. (DOCX) [file pone.0106501.s002.docx]
